# Biogenic silver nanoparticles associated with silver chloride nanoparticles (Ag@AgCl) produced by laccase from *Trametes versicolor*

**DOI:** 10.1186/2193-1801-3-645

**Published:** 2014-10-31

**Authors:** Nelson Durán, Raphael Cuevas, Livia Cordi, Olga Rubilar, Maria Cristina Diez

**Affiliations:** Biological Chemistry Laboratory, Instituto Química, Universidade Estadual de Campinas, CP 6154, CEP 13083-970 Campinas, SP Brazil; Laboratory on Nanostructures Synthesis and Biosystems Interactions (NanoBioss) (UNICAMP/SP), Campinas, SP Brazil; Doctoral Program of Science of Natural Resources, Universidad de La Frontera, Temuco, Chile; Environmental Biotechnology Center Science Nucleus BIOREN, Universidad de La Frontera, Temuco, Chile; Institute of Biology, Universidade Estadual de Campinas, Campinas, SP Brazil; Department of Chemical Engineering, Universidad de La Frontera, Temuco, Chile

**Keywords:** Silver nanoparticles, Silver chloride nanoparticle, Laccase, *T. versicolor*

## Abstract

In the present study, semi-purified laccase from *Trametes versicolor* was applied for the synthesis of silver nanoparticles, and the properties of the produced nanoparticles were characterized. All of the analyses of the spectra indicated silver nanoparticle formation. A complete characterization of the silver nanoparticles showed that a complex of silver nanoparticles and silver ions was produced, with the majority of the particles having a Ag^2+^ chemical structure. A hypothetical mechanistic scheme was proposed, suggesting that the main pathway that was used was the interaction of silver ions with the T1 site of laccase, producing silver nanoparticles with the concomitant inactivation of laccase activity and posterior complexing with silver ions.

## Introduction

The literature has demonstrated that the mechanism used for the formation of metal nanoparticles is the action of a large number of enzymes secreted by fungi in special reductases (Ahmad et al. 2003; Durán et al. 2007; Durán et al. 2011; Jain et al. 2011). Currently, the use of white-rot fungi for the production of metal nanoparticles is a recent branch of research with less than a decade of study (Rubilar et al. 2008; Tortella et al. 2008; Acevedo et al. 2012). The number of reports on synthesis is very limited, and the use of different species of fungi has been limited to a few species, such as *P. chrysosporium* (Vigneshwaran et al. 2009), *Pleurotus sajor caju* (Nithya and Ragunathan 2009), *Coriolus versicolor* (Sanghi and Verma 2009), *Pycnoporus sanguineus* (Chan and Don 2012a) and *Shizophyllum commune* (Chan and Don 2012b). Furthermore, Sanghi et al. (Sanghi et al. 2001) demonstrated that the laccase enzyme secreted by *Phanerochaete chrysosporium* white-rot fungus was responsible for the formation of gold nanoparticles. At the same time, it was reported that a laccase from the ascomycete *Paraconiothyrium variabile* was isolated from soil, and a blue enzyme with laccase activity was purified and characterized (Forootanfar et al. 2011). This enzyme was also able to produce gold nanoparticles (Faramarzi and Forootanfar 2011).

In this context, white-rot fungi isolated from temperate forests in southern Chile have potential for biosynthesizing metal nanoparticles due to their high production of extracellular enzymes, such as laccase and peroxidases (Tortella et al. 2008), which have been shown to biosynthesize metal nanoparticles (Vigneshwaran et al. 2009).

The aim of this study was to evaluate the biosynthesis of silver nanoparticles through the semi-purification of laccase from *Trametes versicolor*, a white-rot fungus, as well as to characterize the nanoparticles by electronic microscopy, analysis of the distribution of size by DLS measurements, stability of the nanoparticles by Zeta potential and, finally, by FTIR and XRD.

## Materials and methods

### Laccase production

Laccase was obtained from *T. versicolor* and semi-purified following a previous published procedure (Cordi et al. 2007; Leonowicz and Grzywnowicz 1981), and the silver nanoparticles were obtained after incubation in the presence of silver nitrate. Briefly: Laccase was obtained from *T. versicolor* (CCT 4521) cultivated for 4-20 days at 30°C while shaking at 240 rpm in liquid medium following Cordi et al. (2007) Laccase production was induced by copper sulfate (0.1 mmol L^-1^) and 2,5-xylidine (1 mmol L^-1^) with 96 h of fungal culture.

### Laccase semi-purification

The purification followed the previously published results of Cordi et al. (Cordi et al. 2007). The culture filtrate from the procedure described above in the presence of 0.1 mmol L^-1^ of copper sulfate (through 380 mesh) was frozen, thawed, filtrated through a Millipore 0.45 mm membrane and lyophilized. A solution containing 2.0 g of lyophilized crude extract, 30 mL of citrate-phosphate and buffer (75 mmol L^-1^, pH 5.0) was precipitated with 90% ammonium sulfate. The precipitate was eluted in a Sephacryl S-200 (Sigma) column using the same buffer as the mobile phase. The fractions containing laccase activity were collected. Another precipitation and elution was performed to eliminate the dark pigments from the filtrate. The fractions with laccase activity were collected and lyophilized. The lyophilized sample was resuspended in 10 mmol L^-1^ (pH 5.0) citrate-phosphate buffer and applied to a column containing DEAE cellulose (Leonowicz and Grzywnowicz 1981). The laccase was eluted with 10 mmol L^-1^ citrate-phosphate buffer, and a NaCl gradient of zero to 1 mol L^-1^ was applied. The fractions obtained were lyophilized and stored in a freezer (Cordi et al. 2007).

### Enzyme activity assay

The reagent syringaldazine was used as a substrate for the spectrophotometric determination of the laccase activity. Briefly: a reaction mixture containing 0.6 mL of enzymatic solution, 0.3 mL of citrate-phosphate buffer 0.05 M (pH 5.0) and 0.1 ml of syringaldazine (1 mM) at a final volume of 1 mL was mixed for 5 min, and the absorption at 525 nm (ϵ_525_ = 65000 M^-1^ cm^-1^) was measured. One laccase unit was defined as the enzyme quantity needed to oxidize 1 mmol of syringaldazine min^-1^ per liter of total enzymatic solution (Cordi et al. 2007).

### Preparation of silver nanoparticles

An aqueous solution of 200 UL^-1^ semi-purified laccase (stock solution of 2300 U L^-1^) was added to an aqueous solution of 1 mmol L^-1^ silver nitrate followed by the incubation of the reaction mixture in the dark at different temperatures (30–50°C). The formation of a brown color indicated the production of silver nanoparticles (Durán et al. 2007).

### Characterization of silver nanoparticles

#### UV-Visible Spectroscopy

UV-Visible spectrophotometer (UV-1650 PC Shimadzu) and Spectronic Genesys GS at a range of wavelength of 200-800 nm was used.

#### X-Ray Diffraction (XRD) Studies

XRD spectra was recorded on a Shimadzu XRD 7000 instrument, and depicted number of Bragg reflections indexed on the basis of the face centered cubic (FCC) structure of metallic silver.

#### Particle size (DLS) analysis and Potential Zeta

The hydrodynamic diameter and zeta potential values of the produced silver nanoparticles were assessed with a Malvern Zetasizer Nanosystem (Worcestershire, UK). The aqueous suspension of the synthesized silver nanoparticles was filtered through a 0.22 μm syringe driven filter unit and the size of the distributed silver nanoparticles were measured by using the principle of dynamic light scattering (DLS) technique made in a Malvern Zetasizer Nano series compact scattering spectrometer.

#### Transmision Electron Microsocopy (TEM)

TEM images of the samples were obtained using a transmission electron microscope (model JEOL JEM 1200EX II), at a Filament; Tungsten Voltage, kV; 40-120 kv. The elemental analysis was carried out in spectrum of energy dispersive X-ray technique.

#### Fourier Transform Infrared Spectroscopy (FTIR)

Characterization of silver nanoparticles was carried out by FTIR using CARY 630 FTIR Agilent Technologies in the range 600-4000 cm^-1^. FTIR reveals the biomolecules responsible for the reduction of silver ions and stabilization of AgNPs in the solution.

#### Scanning Electron Microscopy (SEM)

SEM micrograph was taken using a JEOL 6360LV instrument, 40 kV. The samples were fixed with 2.5% glutaraldehyde overnight at room temperature followed by dehydration with gradient alcohol (10% to 95%) for 20 min and then in absolute alcohol for 2-5 min. The final specimen was prepared by coating the dehydrated sample with monolayer platinum for making the surface conducting.

## Results and discussions

### Laccase production

From the kinetics of laccase production, it was shown that the best laccase activity was reached after 12 days under the studied conditions.

**Figure 1:**
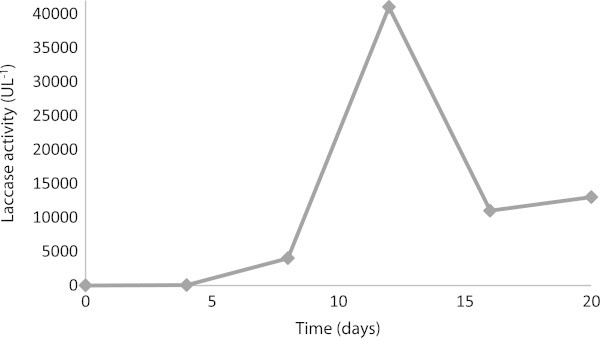
**Laccase activity versus time (days) in the presence of** ***T. versicolor*** **at 0.1 mmol L** ^**-1**^ **of copper sulfate and 1 mmol L** ^**-1**^ **of 2,5-xylidine at 30** ^**o**^ **C (average values of three independent measurements ± 2% error).**

It was previously demonstrated that laccase activity was negligible in the absence of copper sulfate in in this culture (Cordi et al. 2007). The highest laccase activity was observed at 0.100 mmol L^-1^ copper sulfate, and the value was 41,000 U L^-1^ at the 12th day (Figure 1). The laccase semi-purification shows that most of the pigments present in the crude extract were removed by the Sephacryl S-200 step (Table 1). The lower laccase activity compared to the crude extract was most likely due to the purification of isoenzymes. The final fraction from Sephacryl S-200 (Leonowicz and Grzywnowicz 1981) was used in all of the experiments in this study.

**Table 1:** **Extracellular laccase semi-purification from T. versicolor**

Purification (Steps)	Volume (mL)	Total activity (U L ^-1^)	Total protein Content (μg L ^-1^)	Specific activity (U mg ^-1^)	Purification (folds)
Crude extract	900	2700	2500	1	1
Precipitation (NH_4_)_2_SO_4_					
Sephacryl S-200 (1)	60	8600	415	21	10
Precipitation (NH_4_)_2_SO_4_					
Sephacryl S-200 (2)	44	10300	277	37	35

### Reduction of a silver ion solution by laccase

**Figure 2:**
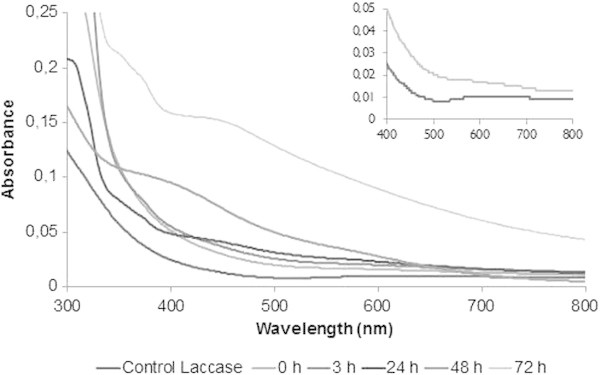
**UV-Visible absorption spectra of silver nanoparticles using a 1 mM AgNO3 solution in the presence of semi-purified laccase from T. versicolor (pH 9.0, 50°C).** The insert shows the UV–vis absorption in the region of 600 nm.

The absorption spectra of an untreated and treated silver solution (Figure 2) indicated the formation of silver nanoparticles due to the presence of the surface plasmon absorption of silver nanoparticles between 420 to 440 nm. The absorption of laccase (T1 site) at 600 nm, after the addition of silver ions, disappeared completely with the concomitant loss of laccase activity (Figure 2, insertion).

The wide plasmonic band of this interaction of silver ions and laccase is most likely an indication that another nanometric species is present. This is discussed later because the XRD shows the formation of silver chloride nanoparticles, in addition to silver nanoparticles, similarly with a plasmonic band at 440-460 nm, as previously described by Gopinath et al. (2013).

**Figure 3:**
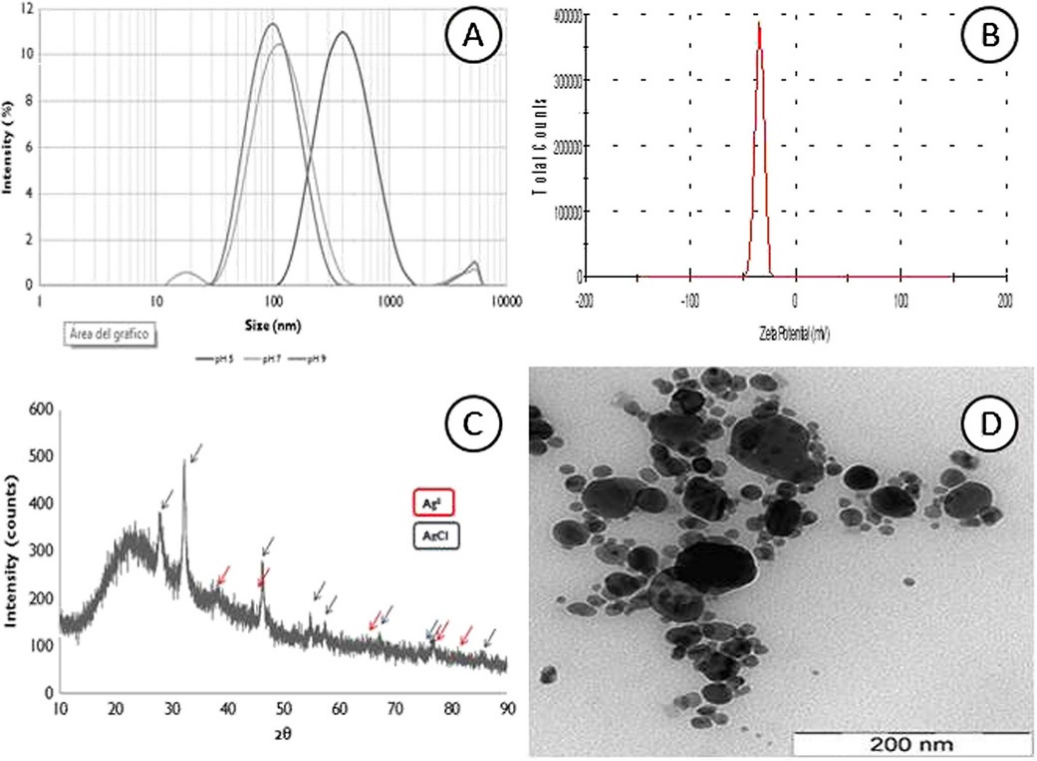
**Characterization of silver nanoparticles. (A)** Dynamic Light Scattering (DLS)*.* **(B)** Zeta potential. **(C)** XRD pattern. **(D)** Transmission electron micrograph (TEM).

The effect of temperature on the size of the silver nanoparticles was also studied and showed a 90 to 370 nm distribution from 30°C to 70°C of silver nanoparticles after 72 h of incubation (data no shown). The nanoparticles produced at 50°C showed particle size distributions of approximately 200 nm at pH 9 (Figure 3).

Figure 3A shows the pH effect on the size distribution of the silver nanoparticles at 50°C (72 h). A similar size of approximately 100 nm at pH 5 and pH 7 was observed. However, at pH 9, an agglomeration occurred, resulting in an increase in the size distribution of up to 200 nm. Figure 3B shows the zeta potential of the silver nanoparticles formed at pH 9 and 50°C (72 h) of -30.0 mV, showing a negative charge distribution of the silver nanoparticle surface.

### Characterization of silver nanoparticles

Figure 3C shows an X-ray diffraction (XRD) pattern that is compatible with the cubic phase of Ag with diffractions points at 38°, 45°, 64.5°, 78° and 81.7° of 2θ, which can be indexed to the (111), (200), (220), (311) and (222) planes of the facet centered cubic (FCC) structure (JCPDS file: 65-2871) that coexists with the cubic phase of AgCl at 27.9°, 32.3°, 46.3°, 55.0°, 57.6°, 67.6°, 74.6°, 76.9°, and 85.7° and that corresponds to the (111), (200), (220), (311), (222), (400), (331), (420), and (422) planes (JCPDS file: 31-1238).

This XRD pattern clearly shows a pattern from the interaction of silver nanoparticles with silver ions (Ag_2_^+^ complex), represented normally as the Ag@AgCl product (Wang et al. 2008). The chlorine ions likely come from the semi-purification of the laccase process.

FTIR measurements (not shown) of silver nanoparticles and their bands were observed at 3440-2325 cm^-1^, corresponding to the NH stretching vibration of primary and secondary amines in the protein molecule. Bands at 1634 cm^-1^, 1541 cm^-1^ and 1384 cm^-1^ are attributed to the C = C and C–H of the methyl group, which are present in protein. The absorption peaks at 1236 cm^-1^ and 1048 cm^-1^ are assigned to the –C–O and –C = O of aromatic acids and esters, respectively. The peak at 1452 cm^-1^ is attributed to the symmetric stretching vibrations of the –COO– groups of amino acid residues. The peak at 1653 cm^-1^ corresponds to the presence of amide I and amide II, which arises due to the carbonyl stretch and -N-H- stretch vibration. These data indicate the presence of protein capping in Ag@AgCl nanoparticles.

A transmission electron microscopy (TEM) image of silver nanoparticles is shown in Figure 3D. The micrographic image confirmed that the synthesized Ag@AgCl nanoparticles were spherical in shape, and the size of the particles was less than 100 nm, which correlates with the DLS measurements.

Then, laccase from *T. versicolor*, a white-rot fungi, showed reductive action for the synthesis of silver nanoparticles. Incubating the semi-purified laccase in the presence of silver ions at different temperatures reduced Ag^+^ to Ag^o^ and interaction with silver ions generated Ag@AgCl nanoparticles. The chloride ions most likely come from the semi-purification of laccase, as previously described. Increasing the incubation temperature from 30°C to 50°C decreased the time for the formation of nanoparticles, and at 50°C, the production of large nanoparticles was achieved within a few minutes. It was also observed that the particle size and the distribution of the obtained nanoparticles were inversely dependent on the temperature over a range of 30 to 50°C. Additionally, there was a pH dependence of the size distribution, and at a lower pH, smaller sized nanoparticles were found.

This finding is interesting because the biogenic synthesis of silver chloride nanoparticles by *Bacillus subtillis* (Paulkumar et al. 2013) and from leaf extracts of *Cissus quadrangularis* Linn was recently published (Gopinath et al. 2013).

### Mechanistic aspects on biogenic synthesis of Ag@AgCl

Because there are no quinones or any other intermediates in this reduction, it must be considered to be a different type of reduction process. There are many possibilities for this reduction, as previously described for other enzymes in the literature. The production of silver nanoparticles by laccase is mostly likely due to the presence of free cysteine as a reducing group. The laccase from *T. versicolor* exhibits one cysteine associated with Cu1-Sγ (Cys-453) laccase and is stabilized by a disulfide bridge to domain 1 (Cys-85–Cys-488), and a second disulfide bridge (Cys-117–Cys-205) connects domains 1 and 2 of laccase (Piontek et al. 2002).

All of the data indicated that cysteine was acting as reductant, as previously described from the amylase generation of gold nanoparticles through Au-S bonds with the retention of the biological activity of the enzyme (Rangnekar et al. 1997). In a similar direction, the comparison of laccase stability profiles at different temperatures during the gold nanoparticle synthesis showed that the formation of gold nanoparticles occurred when the activity decreased, meaning that thiols as reducing functional groups might be responsible for forming gold nanostructures (Faramarzi and Forootanfar 2011). This finding was in agreement with the study of Kalishwaralal et al. (2010), which demonstrated that denaturation of the purified amylase increased the rate of gold nanoparticle biosynthesis by exposing the reductive groups (thiol groups) of cysteine to a gold solution. Similar findings and explanations were reported for silver reduction by urease (Sharma et al. 2013).

Eby et al. (2009) synthesized antibacterial silver nanoparticles in an organic solution using lysozyme as the reducing and capping agent, but no aspect of the mechanism were discussed. In the case of lysozyme silver nanoparticles synthesis, hydroxyl groups in tyrosine residues and amine groups in tryptophan residues were found to be responsible for this production (Kumar et al. 2012).

**Scheme 1:**
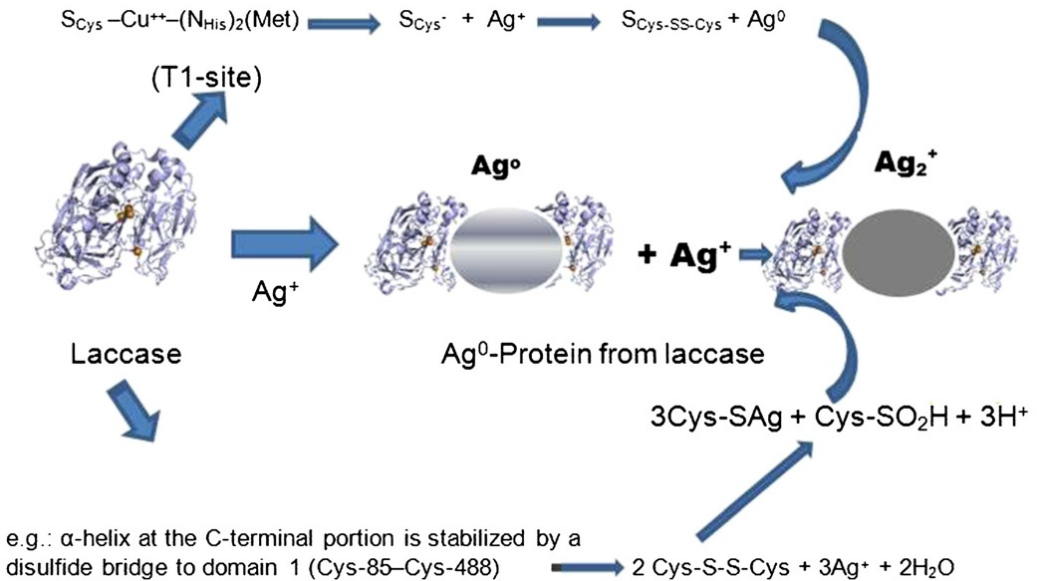
**Hypothetical mechanistic interaction between laccase and silver ions producing silver nanoparticles.**

As previously discussed, in the case of laccase from *T. versicolor,* on the bases of all of the data discussed, two different mechanisms are possible: one involves cysteine associated to Cu type I (T1 site) (sulfhydryl group of cysteine- Cys^-^) in the laccase structure reacting with the silver ion, reducing it to silver nanoparticles while the cysteine anion is oxidized to Cys-Cys in the laccase. The T1 site of laccase imparts a light blue color to the enzyme solutions and is characterized by a distinctly pronounced band of optic absorption at a wavelength of 600 nm (Morozova et al. 2007). This suggested mechanism is possible because, in this study, a rapid disappearance of the absorption band at approximately 600 nm in the *T. versicolor* laccase occurred after the addition of silver ions with complete inactivation of the enzyme (Figure 1 inserted). The second possibility is the direct interaction of silver ions with the Cys-Cys moiety, generating silver nanoparticles, and the sulfhydryl moiety bound to silver nanoparticles as capped proteins (Cecil and McPhee 1957) (Scheme 1).

This interaction of silver ions and the Cys-Cys moiety in the laccase domain most likely inactivates the enzyme, as observed in this study.

This new concept of non-enzymatic catalytic biogenic silver nanoparticles expands the possibilities of the actual syntheses of the biogenic silver nanoparticles (Gade et al. 2008; Bawaskar et al. 2010; Gaikwad et al. 2013; Edmundson et al. 2014; Kashyap et al. 2013; Birla et al. 2013; Tran et al. 2013; Ingale and Chaudhari 2013). The combination of silver chloride nanoparticles, as was detected in this case, associated to silver nanoparticles is another aspect of the great potential of these nanostructures, as shown by previous results of the microbial or photocatalytic activities of silver chloride nanoparticles (Paulkumar et al. 2013; Choi et al. 2008; Min et al. 2010; Naik et al. 2011; Wang et al. 2008).

### Final remarks

The actual data on the biogenic synthesis of Ag@AgCl nanoparticles with laccase demonstrated that the sulfhydryl group is the reducing agent. The most important aspect of this reaction is the sulfur group at the T1 site of laccase activity. The typical absorption band of this blue protein of approximately 600 nm disappeared immediately after the addition of silver ions with the concomitant loss of laccase activity. The interaction with the sulfur-sulfur bridge directly generates the reduction, and binding to the laccase proteic moiety is another possibility. The Ag@AgCl nanoparticles appeared to be a potential antimicrobial in addition to possessing the activities of silver nanoparticles alone.
